# Metabolic acclimation to warming links unexpected immune activation and sexual dimorphism attenuation in *Xenopus tropicalis*

**DOI:** 10.1038/s42003-025-08340-0

**Published:** 2025-06-23

**Authors:** Jiaying Li, Jiahao Zhu, Ayi Budian, Yiwei Zeng, Shouhong Wang, Yuzhou Gong, Xungang Wang, Longhui Zhao, Na Tang, Si Zheng, Rong Han, Songping Zhan, Ting Xie, Ting Chen, Xiangzhen Li, Jianping Jiang

**Affiliations:** 1 Chengdu Institute of Biology, Chinese Academy of Sciences, Chengdu, 610213 China; 2 Department of Automation, Tsinghua University, Beijing, 100084 China; 3 Department of Computer Science and Technology, Tsinghua University, Beijing, 100084 China; 4 Engineering Research Center of Soil Remediation of Fujian Province University; College of Resources and Environment, Fujian Agriculture and Forestry University, Fuzhou, 350002 China

**Keywords:** Zoology, RNA sequencing

## Abstract

Maintaining a dynamic balance among various life-history traits is crucial for survival in a warming world, yet the underlying mechanisms remain enigmatic. In this study, we employed the western clawed frog (*Xenopus tropicalis*) as a model and conducted a long-tern experiment from zygotes to adult stage. We find that even within the previously considered normal temperature range, a 5 °C increase in ambient temperature can establish a new metabolic state, resulting in elevated oxidative stress and a shift in energy allocation towards immune defense at the expense of sexual development. This conceptual framework of temperature-dependent trade-off strategy suggests that, while some studies observed that warm temperature reduces the risk of infection, it is important to note that this change may present challenges in the form of accelerated aging and reduced fertility, especially in ectotherms. These results not only indicate a far more complex adaptive response to future climate change than previously anticipated, but also provide a concise method for constructing animal models to explore diseases related to homeostatic disorders.

## Abstract (web summary)

A transcriptome atlas of Xenopus tropicalis reveals the role of the immune-reproductive trade-off mediated by metabolic reprogramming in maintaining homeostasis from the perspective of inter-organ crosstalk.

## Introduction

Global temperature has broken a new record (https://wmo.int/media/news/july-sets-new-temperature-records) and is predicted to further increase by another 5 °C at the end of this century^1^. Global warming has posed a serious threat to biodiversity, which has increased interest in how organisms counter temperature changes. To maintain whole-body homeostasis and adapt to external conditions, the limited energy resources of organisms must be flexibly invested in growth, development, immunity, and reproduction according to external cues, requiring trade-offs among these life-history traits^2^. The outcomes of these trade-offs are often closely linked to individual fitness and population persistence. Therefore, a comprehensive understanding of the temperature-driven trade-off strategy is essential for predicting the effects of global warming and finding ways to improve physical health.

However, previous studies have often yielded conflicting conclusions, due to potential noise factors (such as diet composition, ultraviolet light, humidity, predators, etc.) in natural systems and short experimental periods (from a few hours to months) in controlled experiments, as well as the limitation of subjects to a single organ or sex^3–5^. Several questions can be raised. For example, what is the fundamental reason behind this paradox? What physiological traits are involved in temperature-driven trade-offs for adaption? How do they interfere with each other? What are the biological signals that trigger the trade-offs? Therefore, a conceptual framework elucidating the temperature-driven adaptive strategy is urgently needed.

**Fig. 1:**
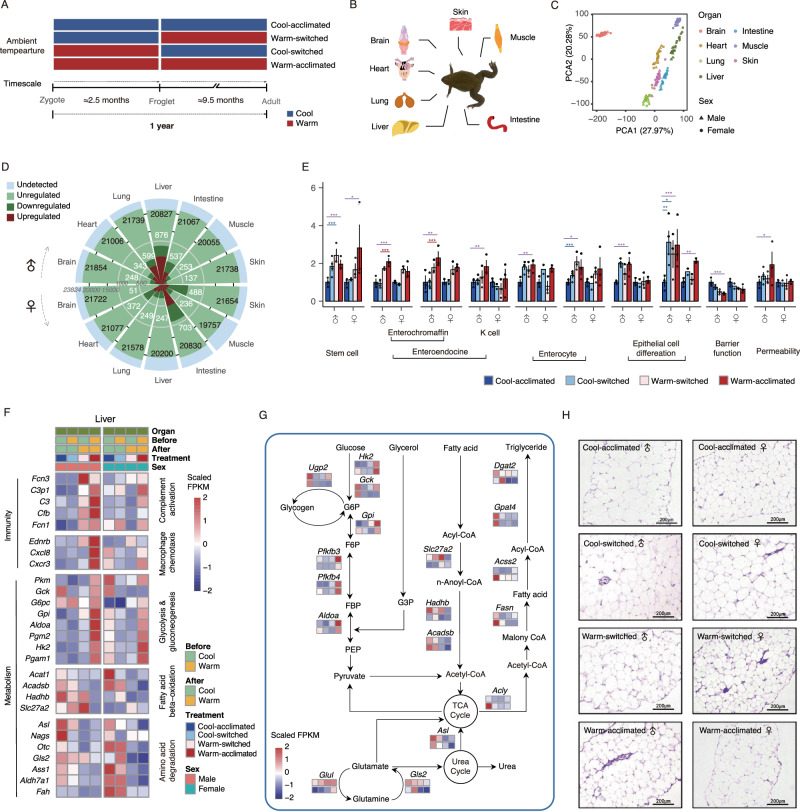
Warm temperature induces an alteration of the energy metabolic state. **A** Schematic diagram of subjected to a temperature reciprocal transfer experiment for 12 months starting at the zygote stage. **B** Schematic representation of the seven organ types for RNA sequencing. **C** Principal component analysis (PCA) based on gene expression. **D** Rose diagrams showing the numbers of DEGs between the Cool-acclimated and Warm-acclimated groups. The first ring (in light blue) refers to undetected transcripts for each organ the numbers in black represent the detected transcripts in each organ type. The second ring (in light green) refers to transcripts unregulated by temperature. The numbers in white present the DEGs significantly regulated by ambient temperature. The innermost ring refers to the number of DEGs in each organ type (dark green, downregulated; dark red, upregulated). *FDR adjusted *p* < 0.05, **<0.01, ***<0.001. Colored lines with asterisks indicate significantly differential pairs (purple, between Warm-acclimated and Cool-acclimated; red, Warm-acclimated involved; blue, Cool-acclimated involved). **E** Relative gene expression of intestinal markers in each temperature-treated group normalized to average expression of the Cool-acclimated groups (♂, male; ♀, female). Colored lines with asterisks indicate a significantly different pair. The data are given as mean ± SEM. **F** Heatmap showing the relative abundance (scaled FPKM) of hepatic DEGs between temperature-switched groups and temperature-acclimated groups, which are related to complement activation, macrophage chemotaxis, glycolysis/gluconeogenesis, fatty acid beta-oxidation, and amino acid degradation, and significantly expressed in at least one condition in male or female. The same color code is used in the entire figure. Significant differential gene expression was detected in at least one condition. **G** Partial metabolic network in liver for differently expressed genes related to glucose, fatty acid, and amino acid metabolism. Boxes from left to right represent the relative abundance of genes in Cool-acclimated, Cool-switched, Warm-switched, and Warm-acclimated groups, respectively. The upper boxes belong to males and the lower boxes belong to females. G6P glucose 6-phosphate, F6P fructose 6-bisphosphate, FBP fructose 1,6-bisphosphate, PEP phosphoenolpyruvate, TCA cycle tricarboxylic acid cycle. **H** H&E staining of adipose tissue of frogs (♂, male; ♀, female) in different temperature-treated groups (scale bars, 200 μm). All experiments were conducted based on six biological replicates in each temperature-treated group with male:female = 3:3 (except Cool-switched group, which is 4:2).

Amphibians are the most threatened vertebrates, with nearly 40% of species now at risk of extinction^6^. Previous studies have shown the acute and subacute effects of extreme temperatures on amphibians^7–9^. However, global warming in nature is a slow and long-term process. Therefore, elucidating the adaptive plasticity of amphibians before environmental temperatures reach extremes is more useful for climate change risk assessment and management. *Xenopus* strains are the most geographically distributed amphibian taxa^10^, widely used in developmental biology, regenerative biology, and genetic research due to their phylogenetic importance and the fact that they possess 80% of human disease genes^11–14^. Here, we used the western clawed frog (*Xenopus tropicalis*) as an example to investigate the temperature-driven trade-off strategy and elucidate the underlying mechanisms from an inter-organ perspective. First, to avoid confounding factors, we established animal models adapted to different temperatures (23 °C, 28 °C) in a controlled environment (Fig. 1A). Then, the vulnerability and resilience to ambient temperatures were measured on multiple organs at morphological, histological, gene expression and transcript alternative splicing levels, following by a comparative analysis with reptiles and mammals. Finally, we identified the signaling molecules that mediate inter-organ communication and illustrated how these signals regulate energy reallocation to achieve physiological trade-offs in response to temperature changes. We presented a conceptual framework that improves our understanding of the temperature-driven adaptive response. The dataset provided in this study will serve as a powerful resource for assessing climate change risks and designing appropriate conservation programs.

## Results

### Warming reprograms energy metabolic states

The phenotypic plasticity reflected by most organ weight was in accordance with the temperature-size rule (i.e., that ectotherms exposed to warm temperatures take shorter to achieve maturity at a smaller size than when exposed to cool temperatures^15^, especially for the organs with high metabolic rate (Fig. S1A, Supplementary Data 1). The small intestine, playing a prominent role in energy intake and allocation, was the most sensitive one in both males and females. To fully assess the impact of ambient temperatures, we performed transcriptomic profiling of 168 samples derived from brain, heart, lung, liver, small intestine, muscle, and skin (Fig. 1B, Supplementary Data 2). Altogether, more than 7000 million reads were generated, and 23,239 genes (involving 23,824 transcripts) were identified from all samples after quality control (Fig. S1B, Supplementary Data 1, 3). Principal component analysis showed that samples were clustered by organ types (Fig. 1C). This ontological dependence, reflected in the unbiased clustering, indicates the accuracy of our experimental protocols.

In line with the organ weight pattern, global transcriptome comparison indicated the highest thermal responsiveness of digestive organs, and revealed that current temperature changes can mitigate the earlier effects to some extent. (Figs. 1D; S1A, C; Supplementary Data 1, 2). The histomorphology results revealed that long-term warm-acclimation reduced the surface area and weakened the muscular layer of the small intestine (Fig. S2A, B; Supplementary Data 1), effects that could attenuate digestion and absorption. After measurement of enteric cell-type-associated genes that reflect differentiation, microbial signals recognition, and physical barrier function^16^, we found that the expression of genes associated with stem cells (*Lgr5*), entero-endocrine system (*Chga*, *Tph1*, *Gip*), and enterocytes (*Maf*, *Nr1h4*) was promoted by warm temperatures (Fig. 1E, Supplementary Data 2). The rapid self-renewal of intestinal cells was further corroborated by the upregulation of *Elf3* and *Klf5* (Fig. 1E, Supplementary Data 2), which is essential for the differentiation and proliferation of small intestinal epithelial cells^17,18^. We detected mutually exclusive expression of *Foxo1* being inhibited, but *Cldn2* being induced by warm temperature in males. *Foxo1* could enhance intestinal barrier function by regulating mucus secretion and tight junction proteins^19^. In contrast, *Cldn2* would lead to barrier loss and increased antigenic permeation^20^. These results suggest that warm temperatures contribute to the rate of intestinal cell renewal and increase intestinal permeability. Overall, the above phenotypic and molecular evidences indicate new energy requirements derived in warm temperatures.

This increased intestinal permeability could lead to higher levels of endotoxins (e.g., lipopolysaccharide derived from gut microbiota) that reach the liver through the portal blood flow, and activate liver complement and macrophages (Fig. 1F, Supplementary Data 2). Many genes involved in glycolysis and gluconeogenesis were upregulated in the warm-acclimated group to provide rapid bursts of energy for immunocyte activation and migration^21^ (Fig. 1F, Supplementary Data 2), whereas the expression of *Ugp2* was downregulated, indicating less glycogen stored (Fig. 1G, Supplementary Data 2). With the reduced oxygen input reflected by less expressed hemoglobin genes in both lung and skin (Fig. S2C, Supplementary Data 1), the lower expression of genes related to amino acid degradation (*Asl*, *Nags*, *Otc*, *Gls2*, *Ass1*, *Aldh7a1*, *Fah*), fatty acid beta-oxidation (*Acat1*, *Acadsb*, *Hadhb*, *Slc27a2*) and triglyceride synthesis pathway (*Acly*, *Fasn*, *Acss2*, *Gpat4*, *Dgat2*) weakened the urea cycle and tricarboxylic (TCA) cycle (Fig. 1F, G; Supplementary Data 2), indicating a decreased utilization of protein and lipid. We made histologic sections of adipose tissue and measured the sizes of the adipocytes to validate the finding at the molecular level (Fig. 1H, Supplementary Data 2). The result showed that smaller adipocytes were more prevalent in the groups with warm temperature at the late stage (Fig. S2D, Supplementary Data 1). In addition, the expression *Dgat2*, the rate-limiting enzyme gene in triglyceride synthesis, was positively correlated with adipocyte size and adipose weight (Fig. S2E, F; Supplementary Data 1). These results suggest that ambient temperature can affect *X. tropicalis* in terms of the preference for nutrient utilization and storage, i.e., warm temperature induces alteration of metabolic state from fatty acid oxidation to glycolysis.

### Warming promotes immune defense and ROS stress, especially in males

**Fig. 2:**
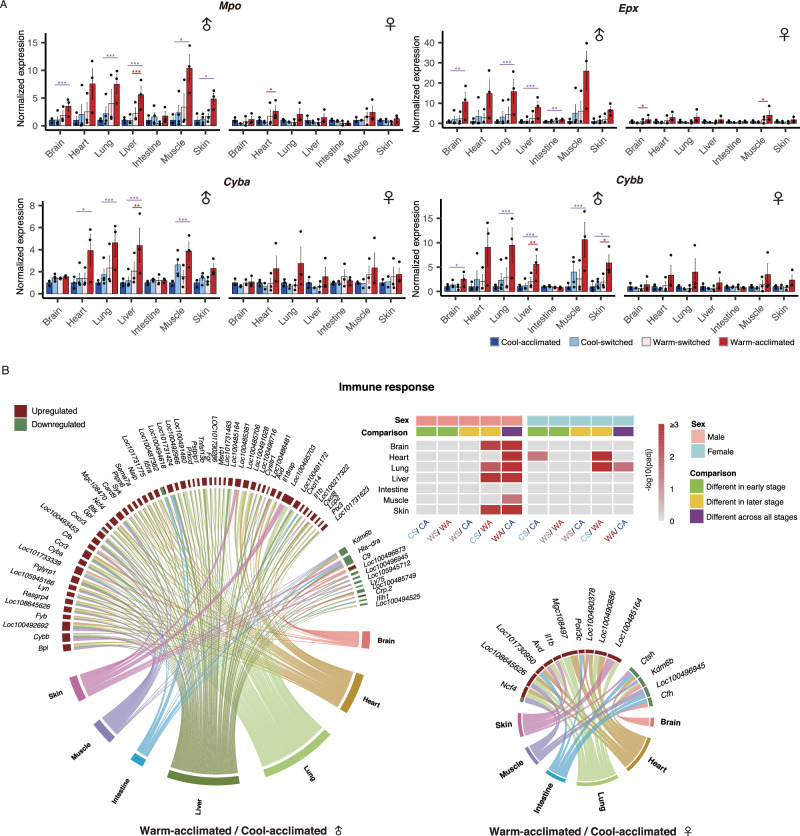
Warming promotes ROS stress and defense response, especially in males. **A** Expression of genes associated with reactive oxygen species biosynthesis in males and females (left, male; right, female). The histograms show the relative abundance in each temperature-treated group normalized to the average expression of the Cool-acclimated group. The data are given as mean ± SEM (|log2 FC| > 1, FDR-adjusted *p-*value < 0.05). **B** Warm temperature activates the immune response. The heatmap (top right) shows the results of GO enrichment analysis between each group. The color bar indicates the FDR-adjusted *p-*value. CA Cool-acclimated, CS Cool-switched, WS Warm-switched, WA Warm-acclimated. Chord diagrams (left and down right) show immune response-related DEGs that were significantly expressed between the warm-acclimated group and the cool-acclimated group (|log2 FC| > 1, FDR adjusted *p*-value < 0.05. left, male; right, female). The length of the brick for each gene corresponds to the sum of |log2 FC| in multiple organs. The length of the brick for each organ corresponds to the sum of |log2 FC| in all related DEGs. *FDR adjusted *p* < 0.05, **<0.01, ***<0.001. Colored lines are described as in FC | >1, FDR adjusted *p*-value < 0.05. left, male; right, female). The length of the brick for each gene corresponds to the sum of |log2 FC| in all related DEGs. *FDR adjusted *p* < 0.05, **<0.01, ***<0.001. Colored lines are described as in Fig. 1. All experiments were conducted based on six biological replicates in each temperature-treated group, with male:female = 3:3 (except Cool-switched group, which is 4:2).

Warm temperature accelerates the metabolic rates of ectotherms due to the thermodynamics of the biochemical reactions^22^. In this study, we found that genes related to peroxidase (*Mpo*, *Epx*) and cytochrome (*Cyba*, *Cybb*) were highly expressed in warm-acclimated males compared with their counterparts in the Cool-acclimated group (Fig. 2A, Supplementary Data 2), and previous studies have elaborated that these genes are strongly interconnected with ROS generation^23^. The accumulation of ROS will cause oxidative damage to various biological molecules, combined with low-grade systemic inflammation and accelerate the aging process^24^, which has been demonstrated in mice and fish^25,26^. Therefore, the increase of ROS generation and systemic inflammation induced by warm temperature in our study (Figs. 2A; S3A; Supplementary Data 1, 2) provides a molecular explanation for the conjecture of Cayuela et al. that global warming promotes amphibian aging, particularly in males^27^. To minimize the damage from ROS, the expression of genes related to antioxidant enzymes (*Cat*, *G6pd*) and DNA repair (*Ogg1*) was significantly upregulated (Fig. S3B, Supplementary Data 1)^28–30^, suggesting that a higher cost in terms of reducing oxidative damage was required in the warm-acclimated group.

The by-product ROS can not only serve as a signal to drive metabolic switch from oxidative phosphorylation to glycolysis, but also supports immune response and pathogen clearance^31^. When summarizing the results of Gene Ontology (GO) enrichment analyses, we found that the responses to ambient temperature are sex-specific (Fig. S4, Supplementary Data 1). For males, terms enriched in multiple organs were associated with immunity (e.g., inflammation response, respiratory burst, innate immune response and regulation of cytokine production), while the effect of ambient temperature on females was primarily in terms of metabolism (e.g., small molecule metabolic process, carboxylic acid metabolic process, and alpha-amino acid metabolic process). Therefore, we hypothesized that sex hormones and related signals might be closely linked to temperature-driven plasticity.

### Warming attenuates sexual dimorphism

**Fig. 3:**
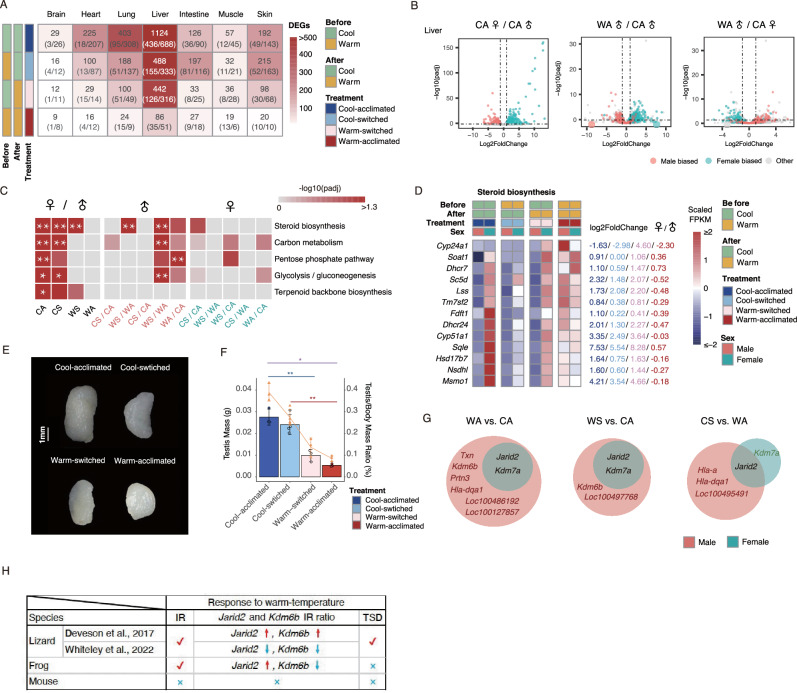
Warm temperature attenuates sexual dimorphism. **A** The numbers of DEGs between male and female in different temperature-treated groups among multiple organs. **B** Warm temperature feminizes gene expression in males but masculinizes gene expression in females. Volcano plot of hepatic sex-biased genes in the Cool-acclimated group (left). Differences in hepatic gene expression between Warm-acclimated males and Cool-acclimated males categorized by sex-biased expression in Cool-acclimated frogs (middle). Differences in hepatic gene expression between Warm-acclimated females and Cool-acclimated females categorized by sex-biased expression in Cool-acclimated frogs (right). Colors indicate male-biased (red), female-biased (green), and unbiased (gray) expressed genes. The dotted lines paralleling to *x* axis or *y* axis indicate |log2 FC| = 1 or −log_10_ adjust *p*-value = −log_10_ 0.05, respectively. CA Cool-acclimated, WA Warm-acclimated. **C** The top 5 hepatic KEGG pathways were significantly enriched between male and female in the Cool-acclimated group. And the enrichment analysis of these corresponding pathways between the indicated contrasts. *FDR adjusted *p* < 0.05, **<0.01, ***<0.001. (male and female in a specific temperature-treated group, left; males in different temperature-treated groups, middle; females in different temperature-treated groups, right; CA Cool-acclimated, CS Cool-switched, WS Warm-switched, WA Warm-acclimated). **D** Heatmap showing the hepatic expression of genes involved in the steroid biosynthesis pathway between temperature-switched groups and temperature-acclimated groups and significantly expressed at least one condition in male or female. Gene expression is shown as the z-score of FPKM (fragments per kilobase of transcript per million mapped reads). The numbers on the right indicate the log2 FC values between male and female in a specific temperature-treated group. Significant differential gene expression was detected in at least one condition. **E** Representative images of testis (unilateral) in each temperature treatment (scale bars, 1 mm). **F** The histogram indicates the weight and the line indicates the relative weight of the testis (testis weight versus body weight) in each temperature treatment. The data are given as mean ± SEM. **G** Venn plots show the shared DEGs between indicated contrasts across multiple organs in males and females. **H** Summary of the relationship between sex phenotype, intron retention, and ambient temperature across lizard, frog, and mouse. The data are presented as mean ± SEM. Colored lines are described as in Fig. 1. All experiments were conducted based on six biological replicates in each temperature-treated group with male:female = 3:3 (except Cool-switched group, which is 4:2).

To assess the relationship between temperature and sexual dimorphism, we compared the number of DEGs between males and females in each temperature-treated group. We found that the number decreased sharply with the increasing temperature, especially in the liver (Fig. 3A, Supplementary Data 2). This indicates that warm temperatures attenuate sexual dimorphism in amphibians at the transcriptomic level. To further examine the variation of these sexually dimorphic DEGs affected by temperature, we defined the genes showing significantly different patterns between males and females in the Cool-acclimated group as male- and female-biased genes, and then checked their differential expression pattern in the warm-acclimated group. We found that the male-biased genes were downregulated in warm-acclimated male frogs, whereas the female-biased genes were upregulated. Conversely, the male-biased genes were upregulated in warm-acclimated females, whereas the female-biased genes were downregulated (Fig. 3B).

To examine the biological significance of these sex-biased DEGs influenced by temperatures, KEGG pathway enrichment analysis was conducted on profiles of liver, as it showed pronounced sexual dimorphism. The result showed that steroid biosynthesis, carbon metabolism, pentose phosphate pathway, glycolysis/gluconeogenesis, and terpenoid backbone biosynthesis were the most significantly enriched in the Cool-acclimated or cool-switched groups (Fig. 3C, Supplementary Data 2). The number of significantly enriched terms decreased with increasing temperature, and even no significant enrichment was detected between males and females in the warm-acclimated group. When comparing individuals of the same sex raised in different temperature environments, these sexually dimorphic DEGs-related pathways in the cool environment were also significantly enriched in males (Fig. 3C, Supplementary Data 2). It is suggested that ambient temperature may have a more critical effect on sex development in male frogs, because sex hormones (such as progesterone, androgen, and estrogen) belong to steroids. Next, when checking the expression of DEGs included in the steroid biosynthesis term, we found that, overall, the impact of warm temperature could lead to the expression of these genes upregulated in male but downregulated in females, and lessen the sexual dimorphic pattern after a life cycle exposure (Fig. 3D, Supplementary Data 2). To further determine the impact of the sex-biased gene expression attenuation on the development of secondary sexual characteristics, we measured testis weight and relative weight (testis weight/body weight) (Fig. 3E, F; Supplementary Data 2), which are widely considered as measures of reproductive investment and sperm competition^32,33^. We observed that warm temperatures exerted profound effects on testis weight. The significant reduction of both testis weight and relative weight corroborated the hypothesis that warm temperature suppressed the reproductive activity of *X. tropicalis*.

The Venn plots showed the overlap of DEGs affected by temperature across multiple organs (Figs. 3G; S5A; 3E, F; Supplementary Data 1, 2). Among these, two demethylase protein genes, *Jarid2* and *kdm6b*, have been reported as mediators of male-to-female sex reversal triggered by high temperature in reptiles (*Alligator mississippiensis*, *Trachemys scripta*, and *Pogona vitticeps*) in terms of expression level or intron retention (IR)^34–36^. For *T. scripta*, Ge et al. validated that the expression of *Kdm6b* was dramatically increased during development and its inhibition by warm temperature would lead to male-to-female sex reversal^35^. As for *P. vitticeps*, although some contradictory results exist at the molecular level about the effects of temperature on the two genes’ IR pattern, it is certain that warm temperature poses consistent effect on these two genes and causes sex reversal from male to female^34,36^ (Figs. S5B;3E, F; Supplementary Data 1). However, in endotherms such as mice, the abundance of these genes is stable to ambient temperature (Figs. S5C;3E, F; Supplementary Data 1). Surprisingly, in our results with *X. tropicalis*, the two genes responded significantly to ambient temperature but in opposite patterns (Fig. S5A, D, E). We found that the expression level and IR ratio of *Jarid2* were significantly induced by warm temperature but the corresponding characteristics of *Kdm6b* were significantly inhibited (Figs. S5A, D, E; 3E, F; Supplementary Data 1, 2). To our knowledge, *X. tropicalis* is currently not considered to be a temperature-dependent sex determination species. Our study indicated a complex but important relationship between these two genes, ambient temperature, and sex determination among amphibians, reptiles, and mammals (Fig. 3H).

### Cholesterol metabolites and secreted proteins mediate inter-organ communication for warm-adaptation

Given the high temperature-sensitivity and central role in life activities, we focused on the liver, which is the main synthesis organ of cholesterol (responsible for nearly 80% of total cholesterol) and its metabolites (e.g., steroid hormones and bile acids). Steroid hormones and bile acids are well-known signal molecules for energy metabolism, sex development, and immune response^37^. Our data demonstrated that multiple genes participating in cholesterol biosynthesis were expressed at a higher level in the livers of females than males, and most of these genes were upregulated after warm acclimation in males but downregulated in females compared with those in the Cool-acclimated group (Fig. S6, Supplementary Data 1). As for the bile acid biosynthesis process, the related genes were significantly depressed in warm-acclimated males, which showed opposite trends to cholesterol biosynthesis-related genes (Fig. S6, Supplementary Data 1). These results implied that overall, the cholesterol biosynthesis process was more active in females than males. To acclimate to warm temperatures, males devoted more cholesterol to synthesizing steroid rather than bile acid, which feminized the metabolic state of males (Figs. S6; 3D; Supplementary Data 1, 2). This finding agreed with our analysis of the attenuation of sexually dimorphic differences induced by warm temperature.

**Fig. 4:**
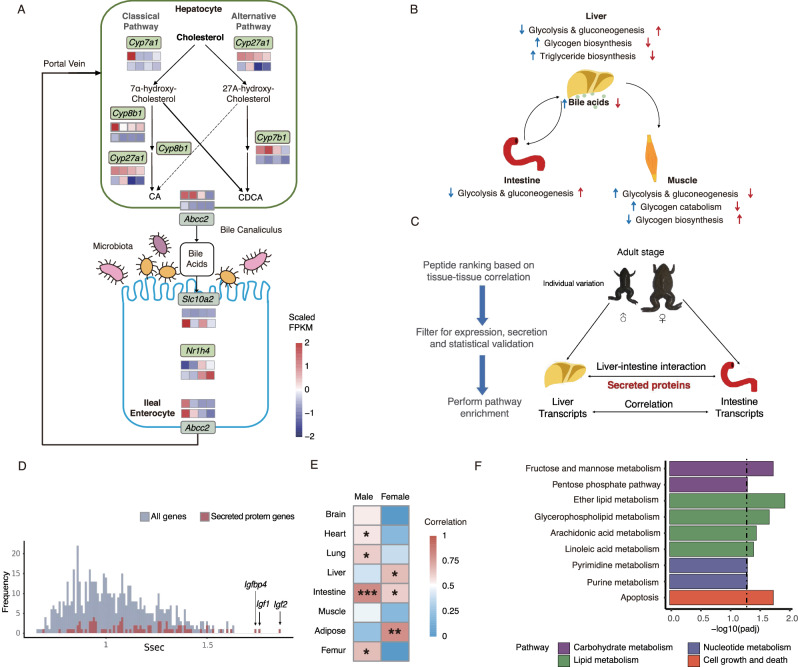
Cholesterol metabolites and secreted proteins play a key role in inter-organ communication for warm acclimation. **A** Partial metabolic network related to the enterohepatic circulation of bile acid. Gene expression is shown as the *z*-score of FPKM. Significant differential gene expression was detected in at least one condition **B** Cross-talk between liver, intestine, and skeletal muscle mediated by bile acid. **C** Generalized steps and schematic framework for identification of the inter-organ cross-talk mediated by secreted protein, and showing the liver-small intestine interaction as an example. **D** Distribution of significance score for all hepatic genes and secreted protein genes across all small intestinal gene expression. Insulin-like growth factor-related genes (indicated by arrow) were identified as top-ranked. **E** Heatmap of correlation between the expression of hepatic Igf1 and organ weight. *FDR adjusted *p* < 0.05, **<0.01, ***<0.001. **F** Distribution of significance score for all hepatic genes and secreted protein genes across all small intestinal gene expression. Insulin-like growth factor-related genes (indicated by arrow) were identified as top-ranked. All experiments were conducted based on six biological replicates in each temperature-treated group with male:female = 3:3 (except Cool-switched group, which is 4:2).

**Fig. 5:**
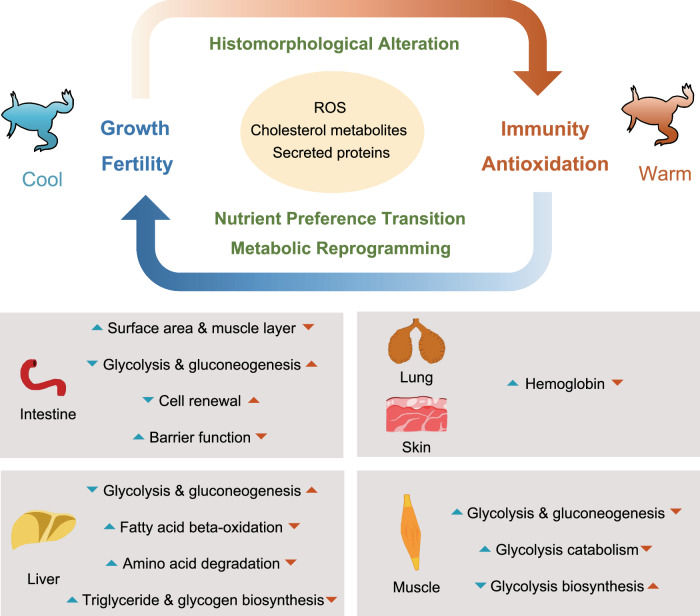
A conceptual framework depicting temperature-driven trade-offs. Warm temperature reduces the intestinal absorption area, increases intestinal permeability, and drives the metabolic state shift from fatty acid oxidation to glycolysis. The metabolic state adapted to warm temperature drives more oxidative stress, resulting in immune activation and the requirement for antioxidation, which weakens the energetic investment for growth and sexual development. The ROS, cholesterol metabolites (e.g., bile acids and steroids), and secreted proteins (e.g., IGF family) serve as molecular signals mediating inter-organ communication to orchestrate the adaptive trade-off strategy.

In detail, we observed that the expression of rate-limiting enzyme encoded genes of the classical pathway (*Cyp7a1*) and alternative pathway (*Cyp27a1* and *Cyp7b1*) of bile acid biosynthesis were both significantly depressed by warm temperature (about 8 to 80 fold) (Fig. S6, Supplementary Data 1). But in mice, cold exposure induced bile acid synthesis by upregulating *Cyp7b1* in the alternative pathway, but did not significantly affect *Cyp7a1* or *Cyp27a1*^38^. These results indicated that the bile acid biosynthetic process in *X. tropicalis* was more sensitive to ambient temperature than in mammals, because at least 75% of the total bile acids are produced by the classical pathway under normal conditions^39^. The bile acids secreted by the liver serve as regulators of gene expression in central and peripheral organs via their cognate receptors^40^. Consistent with mammals^41^, the negative feedback of hepatic bile acids and the expression of bile acid receptors (*Nr1h4*) in the small intestine were observed in *X. tropicalis* (Fig. 5A, Supplementary Data 2). In the warm-acclimated group, the downregulation of bile acid production and upregulation of intestine *Nr1h4* expression reduce the enterohepatic utilization of lipid but promote the utilization of glucose^42,43^ (Fig. S6, Supplementary Data 1). Moreover, we also found the role of bile acids in maintaining glucose homeostasis through negative feedback of regulating glucose metabolism between the liver and skeletal muscle (Fig. S6), which is similar to mammals^42^. Combining the above results, our data suggest that regulating nutrient utilization preference and inter-organ crosstalk through bile acids may be one of the strategies to adapt to different temperatures in *X. tropicalis* (Fig. 4A, B).

Inter-organ crosstalk mediated by secreted proteins is another critical mechanism for maintaining homeostasis^44^. A total of 678 secreted protein genes were identified from our transcriptomic profile. The heatmap and hierarchical cluster analysis revealed that the expression of these genes was organ-specific, and 348 genes were regulated by ambient temperature (Fig. S7A). In order to explore the importance of secreted proteins in mediating inter-organ crosstalk, we employed the Quantitative Endocrine Network Interaction Estimation (QENIE) bioinformatic framework to identify correlations between liver and the small intestine (Fig. 4C). We found that among the secreted proteins, the expression of insulin-like growth factor related genes (*Igf1*, *Igf2*, and *Igfbp4*) were highly correlated with gene expression in the small intestine (Fig. 4D; Supplementary Data 4). *Igf1*, mainly generated in the liver, mediates energetic metabolism and enhances systemic growth^45^. We found that the expression of hepatic *igf1* was significantly inhibited by warm temperature and was significantly correlated with multiple organ weight, particularly with the small intestine (Figs. S7B; 4E; Supplementary Data 1, 2). After performing KEGG enrichment analysis of genes whose expression levels were synergistically changed by the expression of hepatic *igf1* (Supplementary Data 5), we found that pathways associated with carbohydrate metabolism, lipid metabolism, nucleotide metabolism, cell growth and death were significantly enriched (Fig. 4F). These results suggested that the hepatic insulin-like growth factors may serve as regulators coordinating multiple organs to support the trade-off strategy through reprogrammed metabolism.

## Discussion

Mapping the phenotypic and molecular response for thermal adaption across a whole organism is important for understanding the effects of global warming. Previous studies are limited to a few tissues, a single sex, a short time period, or are confounded by uncontrolled noise. Here, based on a high-quality transcriptome atlas of the tropical clawed frog, we identified a wealth of temperature-driven physiological changes within and across organs, including energetic metabolism, antioxidant response, immunity, sexual development, and growth. In addition, the common and species-specific adaptive responses were uncovered by comparative analysis among amphibians, reptiles, and mammals. These results highlight the role of physiological trade-offs regulated by coordination across multiple organs in maintaining homeostasis and environmental adaptation.

There is already a consensus that climate change is leading to a decline in biodiversity. However, it seems paradoxical that warm temperatures have been observed to improve immune response^46,47^. Previous studies have reported that warm temperatures can reduce the prevalence of infections in amphibians^48,49^. However, we must be aware that the immune activation induced by warm temperatures comes at a high cost. On the one hand, we found that warmth induces a new metabolic state that produces more reactive oxygen species, causing oxidative stress with a low inflammatory response, which is the same in both endotherms and ectotherms^49–51^ (Figs. 2A; S3A). Oxidative stress is a driver of ageing^49^. This explains the shortened lifespan of amphibians in the context of a warming climate observed in the field^27^. On the other hand, immune function is highly energy-intensive and must be traded off against other life-history traits, such as gamete production. Previous studies have shown that immunity and testosterone levels are often negatively correlated in male^50^; an overactive immune system can make pregnancy difficult in female^51^. In this study, we found that both sex dimorphism and sex organ mass were significantly reduced in highly immune individuals who adapted to warm environments (Fig. 3). This suggests that the rising temperatures are causing individuals to sacrifice energy expenditure for reproduction to ensure current survival, which is detrimental to population persistence. Many previous studies have ignored this point due to the short exposure period and a few monitoring indices. Therefore, our findings highlight the importance of long-term experiments and an integrated view of the metabolism, immunity, growth, and reproduction of individuals and even the health of future generations when assessing the biological effects of climate change or developing methods for animal conservation.

Dysregulation of homeostasis may lead to various diseases, such as metabolic syndrome and related disorders (characterized by a cluster of metabolic and hormonal disorders disrupting lipid and glucose metabolism pathways)^52^, systemic inflammatory disorders (characterized by dysregulated excessive innate immune responses)^53^, immunological infertility (infertility caused by immunological disorders or a hyperactive immune system)^50,51^, etc. A growing body of evidence suggests that elucidating the mechanisms of inter-organ communication in disease and its mediators is critical to understanding the regulation of homeostasis and the biology of systemic diseases^54^. Western clawed frog is an important model for medical research^13,55^. Our study discovered the molecular signals mediating the energy trade-offs across multiple organs, such as ROS, bile acids, and secreted protein Igf1 etc. ROS drives metabolic switch from oxidative phosphorylation to glycolysis to meet new energy metabolism requirement^31^. The lessened bile acids in a warm environment reduce lipid absorption^42^, induce the overgrowth and translocation of intestinal bacteria^56^, and mediate systemic energy metabolism and immune response through the receptor in distant organs^42^. *Igf1*, mediating systemic glucose metabolic homeostasis^57^ and supporting sexual development and reproduction^58^ as well as suppressing immune inflammation^59^, is highly expressed in a cool environment. Therefore, our results not only provide clues to reveal the nature of amphibian population declines, but also provide a simple and inexpensive method of constructing animal models to explore the above-mentioned diseases. Meanwhile, our results also indicate the necessity of controlling the ambient temperature of experimental animals in medical research. Western clawed frogs are very sensitive to temperature, and the ambient temperatures can significantly affect various physiological functions of them, even within the range considered normal (23–28 °C)^9^. Therefore, in medical research, special attention should be paid to the stability of the ambient temperature to avoid the background noise interfering with the results of physiological experiments.

In this study, using *Xenopus tropicalis* as an example, we present a conceptual framework for the physiological trade-offs caused by temperature changes (Fig. 5). Long-term warming established a new metabolic state, which allocated more energetic resources towards immune defense, but at the cost of reduced sexual dimorphism. Reactive oxygen species, cholesterol metabolites, and hepatic secreted proteins were the key signals mediating the defense-reproduction trade-offs through inter-organ communication. These results explain the molecular mechanisms of amphibians population decline under the scenario of global warming, emphasize the importance of considering the synergies and physiological trade-offs across multiple organs in climate change risk assessment, and also provide models and potential regulatory targets for elucidating the mechanisms underlying complex diseases (such as metabolic syndrome and immune infertility). Future studies will be carried out at different developmental stages (such as larval, metamorphic, and froglet stages) and at the single-cell RNA-sequencing scale to provide more detailed information.

## Materials and methods

### Animal rearing experiment

A pair of adult *X. tropicalis* parents were purchased from Zhejiang University and were acclimated to room temperature with a photoperiod of 12:12 h. The frogs were fed three times daily with fish diet (JBL, Germany; raw protein: 43%; raw fat: 2.5%; raw fiber: 7%; ash: 7%; vitamin A: 25,000 IU kg^–1^; vitamin D3: 2000 IU kg^–1^; vitamin E: 330 mg kg^–1^; vitamin C: 400 mg kg^–1^). The experimental protocols were approved by the Institution of Animal Care and the Ethics Committee of Chengdu Institute of Biology, Chinese Academy of Sciences (CIBDWLL2022007). We have complied with all relevant ethical regulations for animal use.

To reduce the potential interference of host genotypic variation, all individuals surveyed in our study were the offspring of this couple of *X. tropicalis* parents. Breeding was induced using human chorionic gonadotropin. Adult *X. tropicalis* were injected with a dose of 20 U. Twenty-four hours later, the male and female were injected with doses of 150 U and 200 U, respectively, and transferred to a breeding chamber.

All fertilized eggs were collected immediately and then randomly distributed into tanks containing laboratory-prepared water that was then placed in artificial climate boxes set to 23 °C or 28 °C (±1 °C, with humidity = 90% and a 12 h:12 h light/dark cycle). The temperatures were selected according to the optimal temperature range for *X. tropicalis* development as suggested by Khokha et al. ^9^, and temperatures outside of the above range will result in rapid death of the fertilized egg during cleavage stages. Animals were fed with pellets of fish diet (JBL), and the grinding level was adjusted to the size of their jaws. To ensure water quality (pH = 7.31–8.01, nitrite <1.0 mg L^–1^, total NH4^+^ <1 mg L^–1^), aquaculture water was aerated for more than 24 h before use and was renewed every day, combined with tank cleaning. For the sake of even breeding density and avoiding crowding stress, some tadpoles were removed from the tanks as they grew.

To investigate the response of *X. tropicalis* to temperature change, a reciprocal transfer experiment was performed between cool and warm environments when all animals within a group completed metamorphosis. Half of the froglets in cool and warm environments were transferred to their counterpart environment, and half remained in their original environment. Individuals that switched environments were considered as “temperature-switched” groups (warm-switched: Cool–Warm, cool-switched: Warm–Cool), whereas others that remained in their original environment were considered as “temperature-acclimated” groups (Cool-acclimated: Cool–Cool, warm-acclimated: Warm–Warm). Triplicate tanks were randomly used in each temperature treatment. The whole experiment lasted for 1 year (Fig. 1A).

### Sample collection

At the end of the experiment, frogs were euthanized by MS-222 (3-aminobenzoic acid ethyl ester methane sulfonate). The weights of representative organs were measured, including the brain, heart, lung, liver, stomach, small intestine, colon, kidney, adipose, femur, and testis. The gastrointestinal contents were removed before weighing. After measuring the entire length of the small intestine, the adipose tissue and the forepart of the jejunum were stored in 4% paraformaldehyde (Sigma) for light microscopy. Then, the results of the small intestine samples were suspended in RNA buffer (ThermoFisher Scientific) and stored at −80 °C, along with the brain, heart, lung, liver, skeletal muscle, and the skin of the back for RNA sequencing. A single animal is regarded as an experimental unit. All experiments were conducted based on six biological replicates in each temperature-treated group with male:female = 3:3 (except Cool-switched group, which is 4:2). A total of 24 animals participated in the experiment.

### Histology

After being weighed, the adipose tissue and the forepart of the jejunum were fixed by 4% paraformaldehyde (Sigma), paraffin-embedded, cut into 5 μm-thick sections, and stained with hematoxylin-eosin using standard protocols. Briefly, the sections were dried on glass microscope slides, deparaffinized in xylene, and then hydrated in a graded alcohol series (100%, 100%, 95%, 90%, 80%, 70%) and washed with distilled water. Then, the sections were incubated in hematoxylin, washed with running tap water, differentiated in 1% acid alcohol, and again washed with running tap water. After being counterstained with eosin, the sections were dehydrated in a graded alcohol series (80%, 80%, 90%, 90%, 100%) and xylene. Lastly, the sections were mounted with neutral gum. Images were obtained using a digital three-lens camera microscope (BA400Digital). The pictures were analyzed using Fiji. The perimeter, villus length, and wall thickness of the small intestine were measured along with the area of adipocytes.

### Transcriptome library preparation and sequencing

Total RNA was isolated from the brain, heart, lung, liver, small intestine, skeletal muscle, and skin. RNA quality was validated with a BioAnalyzer (all samples had RIN >8). mRNA was purified from total RNA using poly-A oligo-attached magnetic beads. Sequencing libraries were generated using a NEBNext® Ultra™ RNA Library Prep Kit for Illumina® (#E7530L, NEB, USA) according to the manufacturer’s recommendations. After internal size assessment, the libraries were sequenced on an Illumina platform, and 150 bp paired-end reads were generated.

### Gene expression profiling

After quality control and adaptor trimming using fastp software (version 0.23.1)^60^, the reads were mapped to the *X. tropicalis* genome (version 10.0) with HISAT2 software (version 2.1.0)^61^. Read counts for each gene in each sample were performed by HTSeq (version 0.6.0)^62^, and FPKM (Fragments Per Kilobase Million Mapped Reads) was then calculated to estimate the expression levels of genes in each sample. DESeq2 (1.20.0)^63^ was used to identify genes with differential expression in different temperature-treated groups. Genes with |log_2_ fold-change| > 1 and *p-*adj < 0.05 were identified as differentially expressed genes (DEGs).

### Function and pathway annotation

The GO and KEGG (Kyoto Encyclopedia of Genes and Genomes, http://kobas.cbi.pku.edu.cn/)^64^ enrichment of DEGs was implemented by the hypergeometric test, in which the *p*-value is calculated and adjusted by multiple comparisons as a *q*-value (genes in the whole genome were used as a data background when performing enrichment analysis). The terms with *q* < 0.05 were considered to be significantly enriched.

### Intron retention analysis

To compare the expression and splicing pattern of *Jarid2* and *Kdm6b* across amphibian, reptile, and mammalian, we downloaded public data of *P. vitticeps* (NCBI with accession numbers PRJEB5206 and PRJNA699086) and *M. musculus* (NCBI with accession number PRJNA813534) treated by different temperatures. After quality control and adaptor trimming as mentioned above, sequences of each species were aligned to their genome using STAR software (v2.7.0f)^65^ with splice-aware alignment, refer to the accompanying Ensembl gene annotation (*X. tropicalis*: UCB_Xtro_10.0.107, https://www.xenbase.org/xenbase/static-xenbase/ftpDatafiles.jsp; *P. vitticeps*: pvi1.1.100, https://www.ncbi.nlm.nih.gov/assembly/GCF_900067755.1/; *M. musculus*: GRCm39.107, https://www.ncbi.nlm.nih.gov/assembly/GCF_000001635.27/). Parameters are set to output only unique alignments.

Differentially retained introns were detected in each species under different temperatures in order to conduct an unbiased analysis of splicing patterns. The IRFinder (v3.1) software^66^ was utilized to analyze the sequence library, generating a list of intron retention intervals with corresponding information. Candidate intron retention intervals were filtered based on the following criteria: intron retention ratio >0.5, interval coverage >0.9, and intron depth >1.0. Intron retention intervals were identified in the genes *Jarid2* and *Kdm6b* across the three species. Intron retention ratios of each sample from the analysis results were displayed in bar plots and reported as mean ± SEM.

### Inter-organ crosstalk regulator identification

The communication between two organs varies in a coordinated manner. Differences in the expression of a secreted protein in one organ are associated with response genes in the target organ. To identify the secreted protein genes, the bicorrelation coefficient and corresponding *p*-value were calculated for all secreted and response gene pairs with respect to different temperatures using the bicorAndPvalue function from the WGCNA^67^ package. *P*-value matrices for secreted protein genes were ranked based on the significance level of association with the target organ, which was determined by the sum of the significance levels of all response genes. A filtering of the ranking results was performed to ensure that the secreted genes were primarily expressed by the origin organ. To analyze the regulatory role of a secreted protein gene, target genes with bicorrelation coefficients >0.5 and a *p-*value < 0.05 were selected for KEGG pathway and gene pathway enrichment analysis^64^.

### Statistics and reproducibility

The data were presented as mean ± SEM unless otherwise noted. The means of morphological and histological characteristics between groups were compared using one-way analysis of variance (ANOVA), assuming equal variance. The cool- or warm-acclimated groups were used as benchmarks for comparison with the other three groups (i.e., Warm–acclimated vs. Cool–acclimated; Cool-switched vs. Cool–acclimated, Warm-switched vs. Cool–acclimated, Warm-switched vs. Warm–acclimated, and Cool-switched vs. Warm–acclimated). All experiments were conducted based on six biological replicates in each temperature-treated group with male:female = 3:3 (except Cool-switched group, which is 4:2). A two-tailed Student’s *t* test was performed in R software (version 4.2.0) to calculate the *p-*value. The Holm–Bonferroni method was applied to correct the *p-*value with false discovery rate, and an adjusted *p* < 0.05 was considered statistically significant (*, **, and *** indicate *p* < 0.05, *p* < 0.01, and *p* < 0.001, respectively).

### Reporting summary

Further information on research design is available in the Nature Portfolio Reporting Summary linked to this article.

## Supplementary information

Supplementary Information

Supplementary Data 1

Supplementary Data 2

Supplementary Data 3

Supplementary Data 4

Supplementary Data 5

Description of Additional Supplementary Files

Reporting Summary

## Data Availability

All data and materials generated in this study are available in the main text and supplementary materials. Source data for all graphs can be found in the Supplementary Data. All sequencing data have been deposited at the NCBI Sequence Read Archive under accession no. PRJNA956801 and GSA under accession no. PRJCA016668.
