# Supplementary material for: Metabolic acclimation to warming links unexpected immune activation and sexual dimorphism attenuation in Xenopus tropicalis
Source: Commun Biol. 2025 Jun 23;8:952. doi: 10.1038/s42003-025-08340-0 (PMC12185709; doi:10.1038/s42003-025-08340-0)
Supplement: Supplementary file 1 — Supplementary Information [file 42003_2025_8340_MOESM1_ESM.pdf]

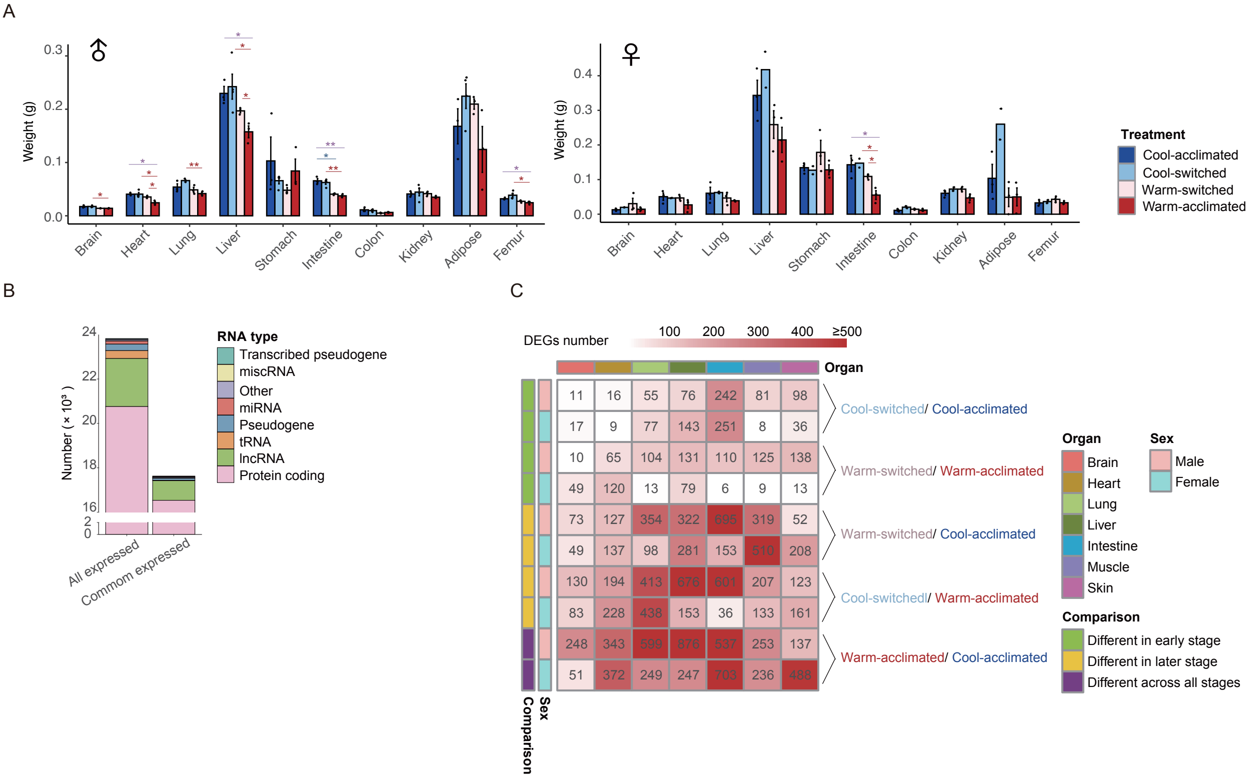

11	16	55	76	242	81	98
17	9	77	143	251	8	36
10	65	104	131	110	125	138
49	120	13	79	6	9	13
73	127	354	322	695	319	52
49	137	98	281	153	510	208
130	194	413	676	601	207	123
83	228	438	153	36	133	161
248	343	599	876	537	253	137
51	372	249	247	703	236	488

Figure. S1 Global alteration induced by ambient temperature.
A Organs weight in adult stage (left, male; right, female) after different temperature treatment. The data are mean ± SEM. **B** Number of transcripts expressed in at least one organ type (23,824, left) or present in all the organ types (17,629, right). Different colors indicate the transcript types. **C** Summary of DEGs between temperature-switched groups and temperatureacclimated groups in each organ type. * FDR adjusted p < 0.05, ** < 0.01, *** < 0.001. Colored lines are described as in Figure. 1.

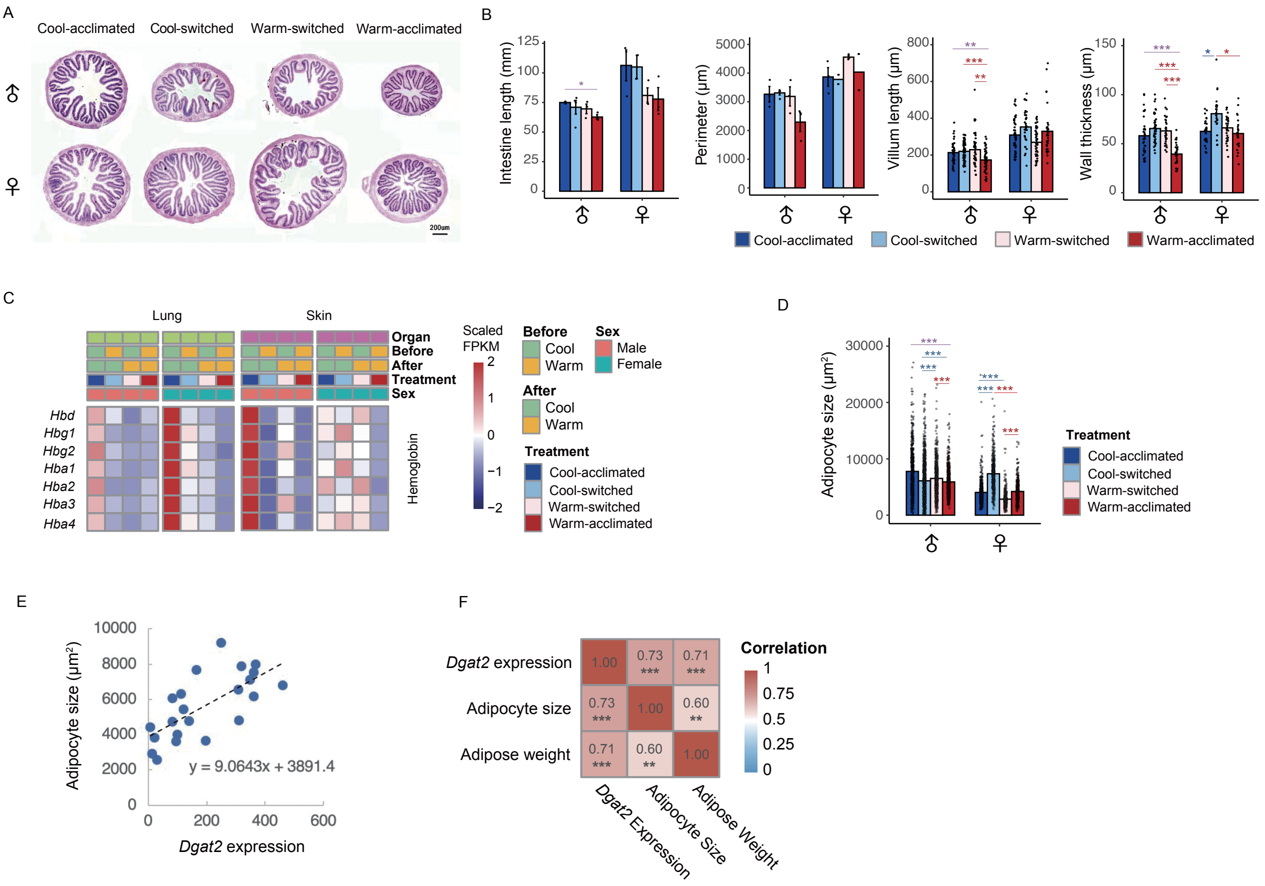

Figure. S2 Warm temperature reduces energy absorption and storage.
A H&E staining of jejunum of frogs (♂, male; ♀, female) in different temperature-treated groups (scale bars, 200 μm). **B** Morphological quantification of intestine length (the first), duodenal perimeter (the second), villus length (the third) and muscle layer thickness (the fourth) in different temperature-treated groups. The data are presented as mean ± SEM. **C** Heatmap of DEGs associated with hemoglobin and defense response to the bacterium in lung and skin. **D** Quantification of adipocyte size in different temperature-treated groups. The data are presented as mean ± SEM. **E** Linear regression of adipocyte size and Dgat2 expression. **F** The Spearman correlation of adipose weight, adipocyte size and Dgat2 expression. * FDR adjusted $p < 0.05$, ** < 0.01, *** < 0.001. Colored lines are described as in Figure. 1.

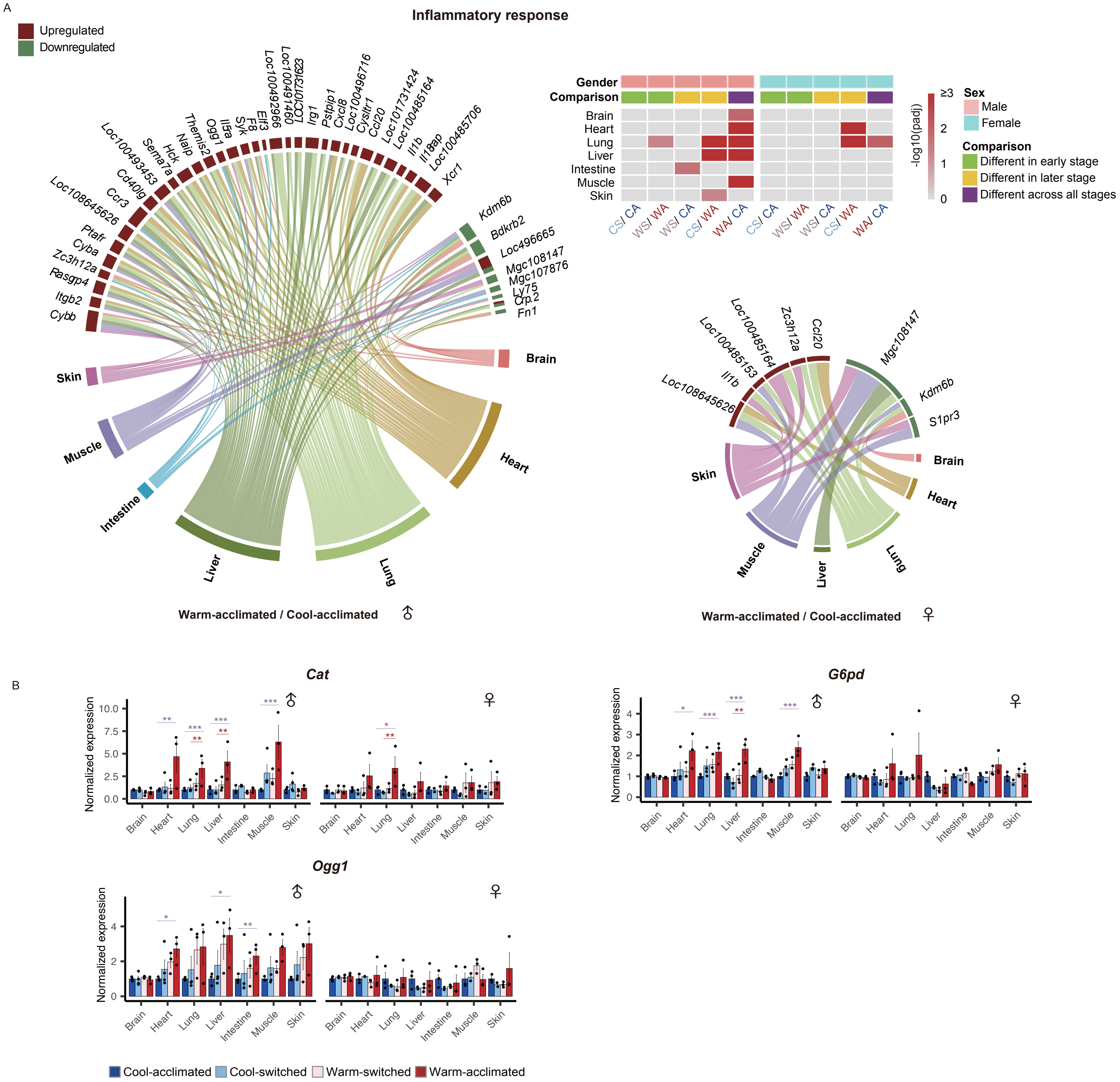

Figure. S3 ROS stress caused by warm temperature promotes antioxidant and inflammatory response.
A ROS stress induces inflammatory response. The heatmap (top right) shows the results of GO enrichment analysis between each groups. The color bar indicates the FDR adjusted p value. (CA, Cool-acclimated; CS, Cool-switched; WS, Warmswitched; WA, Warm-acclimated). Chord diagrams (left and down right) show inflammatory response related genes that significantly expressed between warm-acclimated group and cool-acclimated group (llog2 FCl >1, FDR adjusted p value <0.05. left, male; right, female). The length of the brick for each for each gene corresponds to the sum of llog2 FCl in multiple organs. The length of the brick for each organ corresponds to the sum of llog2 FCl in all related DEGs. Only the genes significantly regulated in more than two organ types are shown. **B** Expression of DEGs associated with antioxidants in males and females (left, male; right, female). The histograms show the relative abundance in each temperature-treated groups normalized to the average expression of the cool-acclimated group. The data are given as mean ± SEM. llog2 FCl, FDR adjusted p value and colored lines are as in Figure.1.

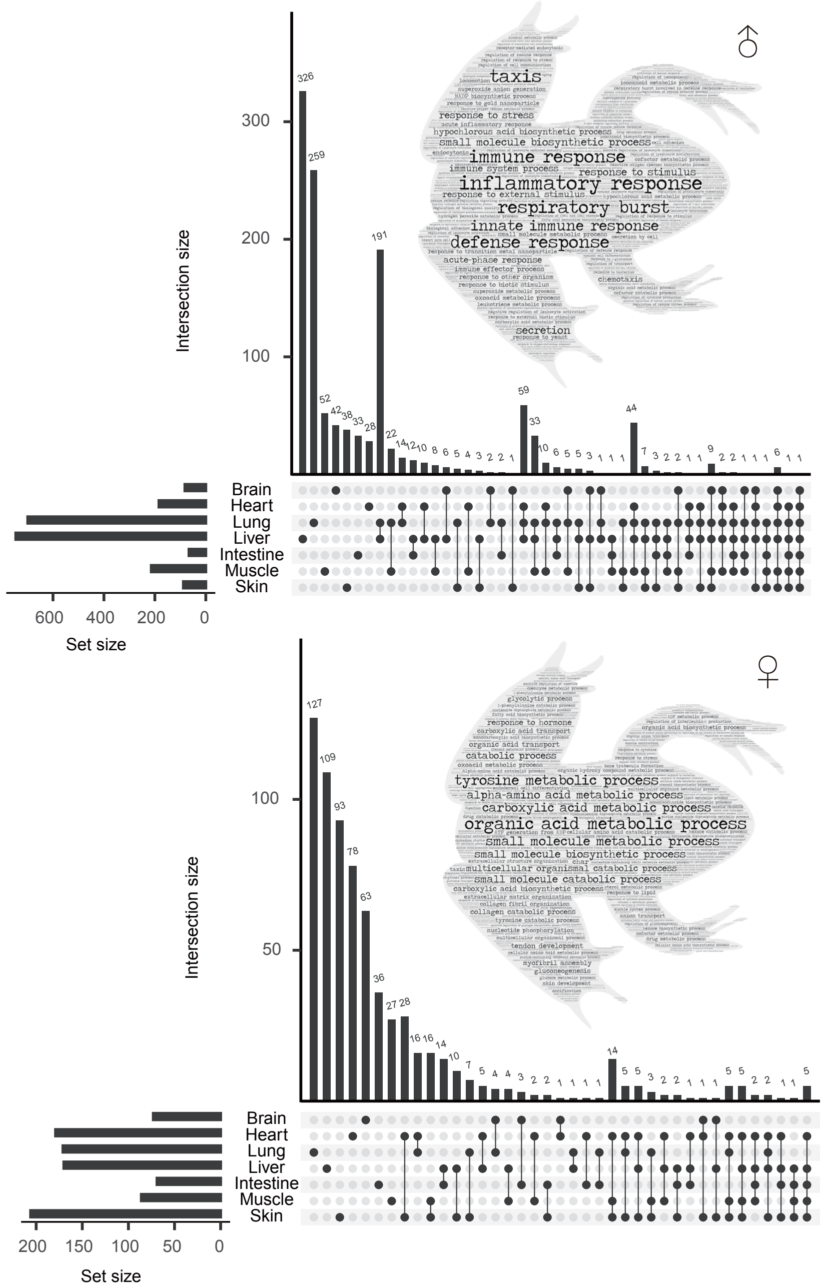

Figure. S4 Summary of significantly enriched GO terms across multiple organ types in males and females.
The word clouds (top) are comprised of words gathered from significantly enriched ontology terms of “biological process” category across multiple organ types respond to different temperature-treatment. The size of words refers to the frequency of the corresponding terms. The upset plots (bottom) show the organ-specific or multi-organ shared terms among organ types.

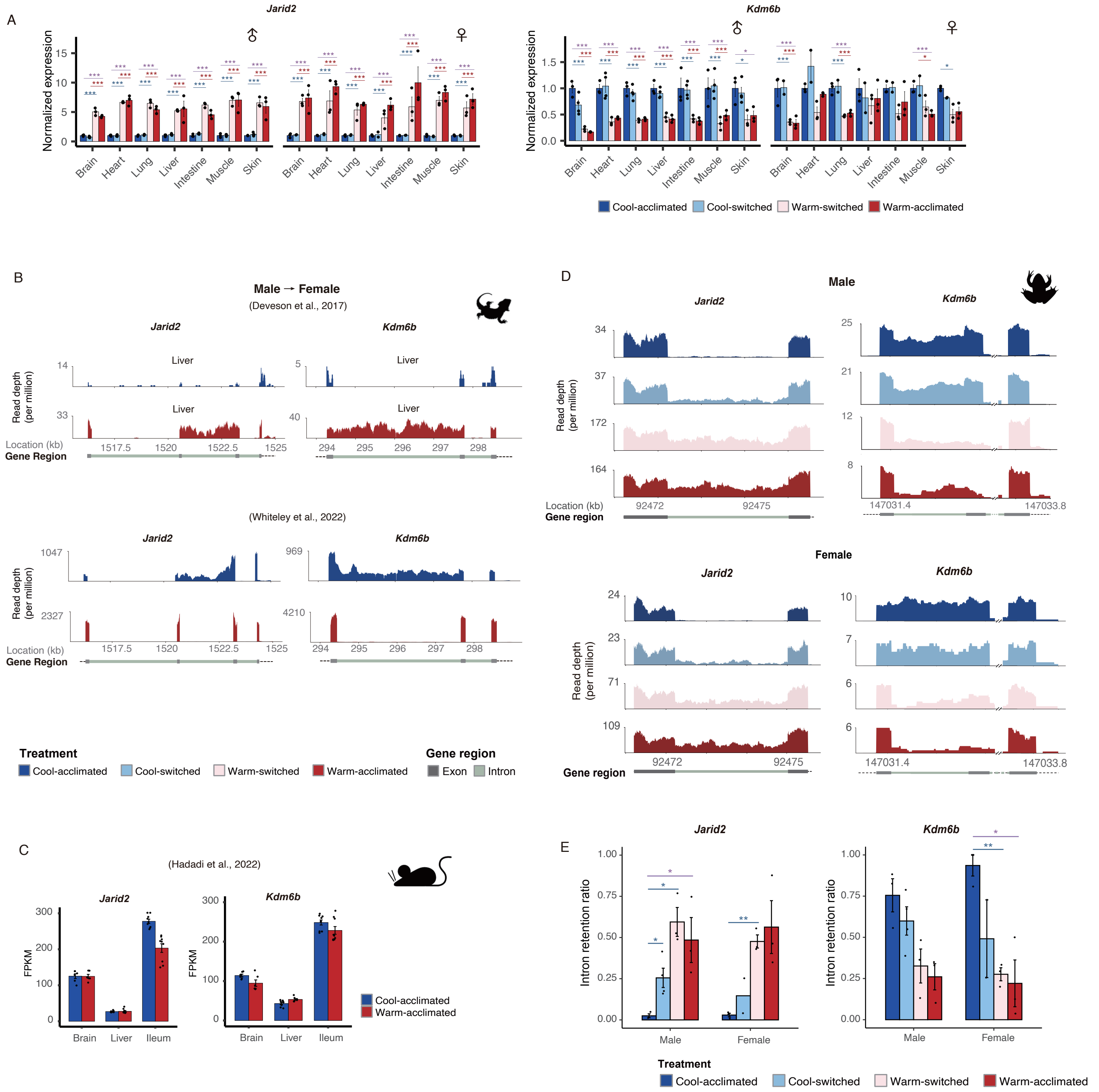

Figure. S5 The response of *Jarid2* and *Kdm6b* to ambient temperature in frog, lizard and mouse.

A Relative abundance (FPKM) of Jarid2 and Kdm6b across multiple organ types in different temperature-treated groups (male, left; female, right). The data are given as mean ± SEM. **B** Temperature-dependent intron retention event of *Jarid2* and *Kdm6b* in lizard. **C** The expression of *Jarid2* and *Kdm6b* in brain, liver and ileum of male mice after 22°C and 34°C exposure for 4 weeks. The data are given as mean ± SEM. **D** Intron retention event of *Jarid2* and *Kdm6b* in frog (dark gray, exon; light gray, intron). Normalized coverage by mapped RNA sequencing reads. **E** The intron retention ratio of *Jarid2* and *Kdm6b* in frog liver under different temperature treatment. The data are given as mean ± SEM. * FDR adjusted $p < 0.05$, ** < 0.01, *** < 0.001. Colored lines are as in Figure.1.

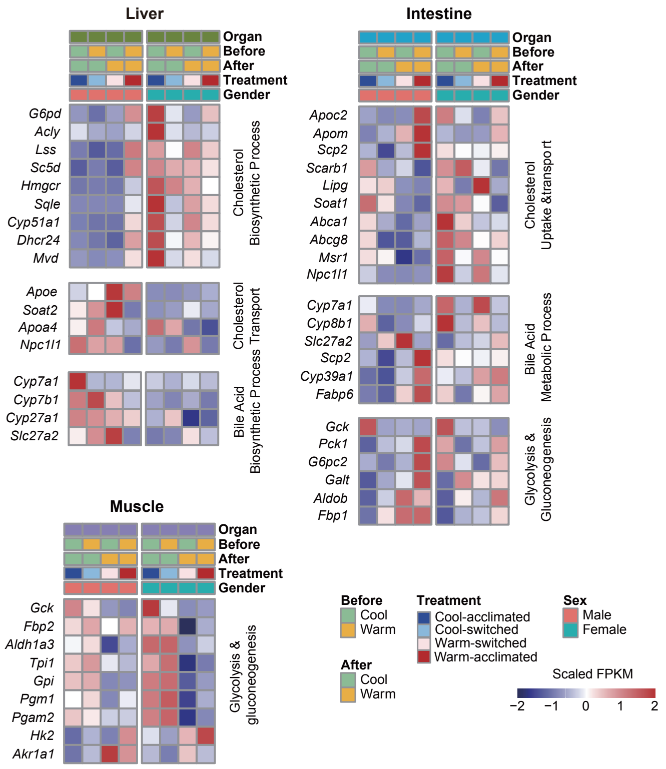

Figure. S6 Gene expression in liver, intestine and muscle.
Heatmap of DEGs related to cholesterol and bile acid metabolism in liver and DEGs related to glycolysis/gluconeogenesis, cholesterol and bile acid metabolism in intestine. Gene expression shown as the z-score of FPKM. Same color code is used in the entire figure.

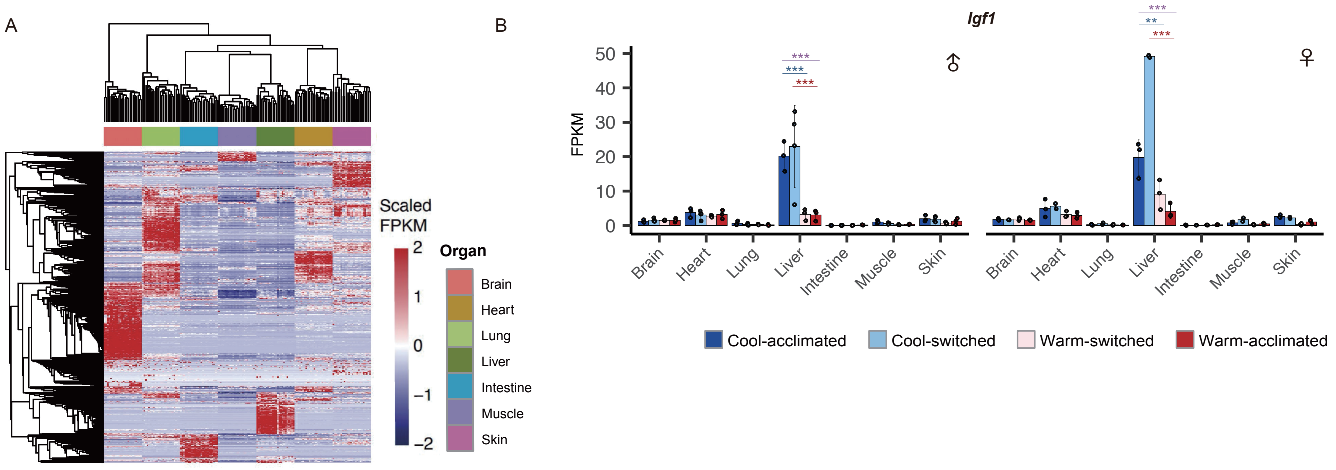

Figure. S7 Expression of secreted protein genes.

A Heatmap of secreted protein genes' expression across multiple organ types. Gene expression shown as the z-score of FPKM. **B** The expression of Igf1 across multiple organ types in male and female (left, male; right, female). The histograms show the relative abundance in each temperature-treated groups normalized to the average expression of the Cool-acclimated group. The data are given as mean ± SEM (|log2 FC| >1, FDR adjusted p value <0.05). * FDR adjusted p < 0.05, ** < 0.01, *** < 0.001. Colored lines are described as in Figure. 1.
