# Supplementary material for: Metabolic acclimation to warming links unexpected immune activation and sexual dimorphism attenuation in Xenopus tropicalis
Source: Commun Biol. 2025 Jun 23;8:952. doi: 10.1038/s42003-025-08340-0 (PMC12185709; doi:10.1038/s42003-025-08340-0)
Supplement: Supplementary file 7 — Description of Additional Supplementary Files [file 42003_2025_8340_MOESM7_ESM.docx]

Description of Additional Supplementary Files

**File name:** Figure. S1

**Description:** Global alteration induced by ambient temperature.

**File name:** Figure. S2

**Description:** Warm temperature reduces energy absorption and storage.

**File name:** Figure. S3

**Description:** ROS stress caused by warm temperature promotes antioxidant and inflammatory response.

**File name:** Figure. S4

**Description:** Summary of significantly enriched GO terms across multiple organ types in males and females.

**File name:** Figure. S5

**Description:** The response of Jarid2 and Kdm6b to ambient temperature in frog, lizard and mouse.

**File name:** Figure. S6

**Description:** Gene expression in liver, intestine and muscle.

**File name:** Figure. S7

**Description:** Expression of secreted protein genes.

**File name:** Supplementary Data 1

**Description:** The source data for figures S1-7.

**File name:** Supplementary Data 2

**Description:** The source data for figures 1-3.

**File name**: Supplementary Data 3

**Description:** A table summarizing the 168 samples included in the study.

**File name:** Supplementary Data 4

**Description:** Distribution of significance score for secreted protein genes in origin organs to target organs.

**File name:** Supplementary Data 5

**Description:** The correlation of secreted protein gene Igf1 in liver and all genes in other organs.
